# Directional sound beam emission from a configurable compact multi-source system

**DOI:** 10.1038/s41598-017-16792-6

**Published:** 2018-01-18

**Authors:** Jiajun Zhao, Rasha Al Jahdali, Likun Zhang, Ying Wu

**Affiliations:** 1 King Abdullah University of Science and Technology (KAUST), Division of Computer, Electrical and Mathematical Sciences and Engineering, Thuwal, 23955-6900 Saudi Arabia; 2 GOWell International LLC, Houston, Texas 77041 USA; 3 National Center for Physical Acoustics and Department of Physics and Astronomy, University of Mississippi, University, Mississippi, 38677 USA

## Abstract

We propose to achieve efficient emission of highly directional sound beams from multiple monopole sources embedded in a subwavelength enclosure. Without the enclosure, the emitted sound fields have an indistinguishable or omnidirectional radiation directivity in far fields. The strong directivity formed in the presence of the enclosure is attributed to interference of sources under degenerate Mie resonances in the enclosure of anisotropic property. Our numerical simulations of sound emission from the sources demonstrate the radiation of a highly directed sound beam of unidirectional or bidirectional patterns, depending on how the sources are configured inside the enclosure. Our scheme, if achieved, can solve the challenging problem of poor directivity of a subwavelength sound system, and can guide beam forming and collimation by miniaturized devices.

## Introduction

Radiation directivity, as one of the important characteristics of a sound source system, describes the quality of a collimated acoustic beam. Sound radiation with high directivity is important in many areas including loudspeaker design, room acoustics, medical ultrasonics, and psychoacoustics^1–5^. However, directivity of acoustic sources at low frequencies in free space or in a subwavelength sound system is always poor^6^.

To achieve high radiation directivity, traditional methods have adapted to use a large horn mouth to confine the sources’ radiation space or use a long phased array to incur wave interference have been adapted. Recently, with the rise of acoustic metamaterials, controlling directivity or steering acoustic beams has been discussed in various schemes, such as grating diffraction^7,8^, band structure engineering^9–14^, transformation coordinates^15^, acoustic metasurfaces^16,17^, and dual metastructures^18^. These schemes modify either boundary conditions, resonances, or the entire propagation region to adjust sources’ directivity. Even so, the entire sound system in these studies is much larger than the working wavelength. Such a large overall scale limits the capability of device miniaturization.

There are a few recent attempts on achieving efficient emission of directional sound waves from a miniaturized subwavelength source. Zhao *et al*^19^. proposed enhancing multipole source emission by using a deep-subwavelength cavity of degenerate Mie resonances to enclose the source, whose radiation pattern and directivity is preserved. The degenerate Mie resonances were caused by the super anisotropy of the enclosure, which cannot be inferred from the previous isotropic model^20^. A latest attempt imitated a dipolar field from a monopole source enclosed in a subwavelength structure of hybrid resonances^21^. In all of these studies, the source-structure system has a limitation on the flexible adjustment of the radiation directivity. Efficient emission of configurable and highly directional sound beams from a subwavelength acoustic system remains as a challenging task.

In this paper, we propose to achieve enhanced radiation directivity of acoustic sources by encompassing configurable multiple sources inside an enclosure. The enclosure contains several monopole sources at low frequencies that radiate otherwise sound of indistinguishable directivity in free space. By deploying the sources inside the enclosure, we numerically demonstrate the formation of directivity enhancements such as unidirectional and bidirectional beam forming. Such directivity enhancements by our configurable sound system are attributed to the interplay between wave interferences and degenerate Mie resonances in the subwavelength space. The configurability of our setup for achieving different types of directivity patterns can be achieved by customized source layouts.

## Results

### Unidirectional Beam Forming

**Figure 1:**
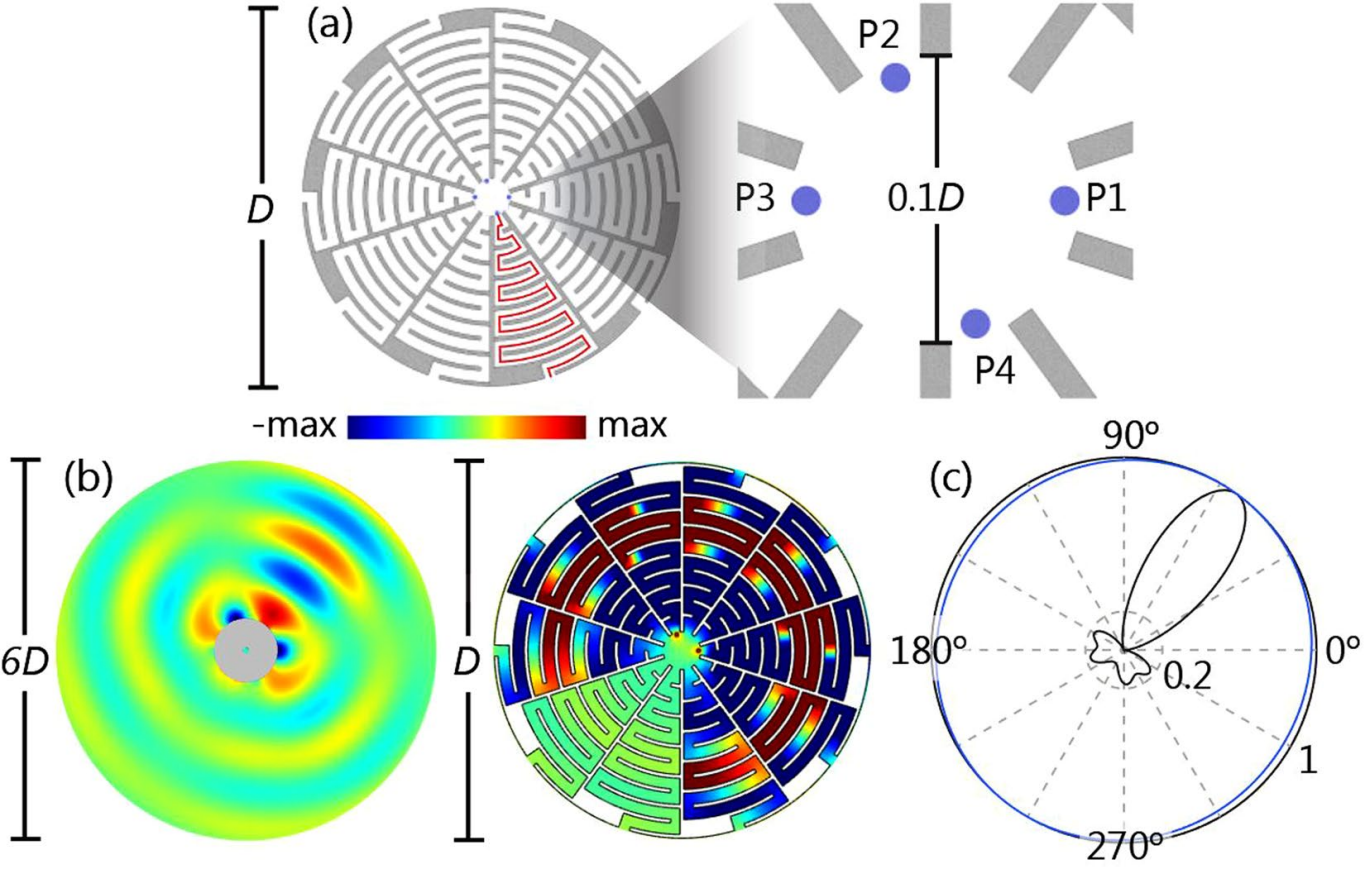
(**a**) A circular enclosure (left panel) made of rigid materials (gray) has coiled air-filled channels with equal sound path (red line), and the outer and inner diameters *D* and 0.1*D*. In the interior region (right panel), there are four positions for sources (blue dots: P1, P2, P3, P4). (**b**) With the in-phase sources at P1 and P2 encompassed by the enclosure, airborne sound of wavelength 1.14*D* propagates unidirectionally, shown by the simulated acoustic pressure. (**c**) The normalized far-field directivity with the enclosure (black curve) and in free space (blue curve). In the presence of the enclosure, the directivity maximum (black curve) is enhanced by 4.8 dB from the radiated sound field without the enclosure. Other parameters are the source diameter 0.01*D*, sound speed in air *c*_air_ = 343 m/s, and the density of air *ρ*_air_ = 1.21 kg/m^3^.

The enhanced directivity can be achieved by the enclosure illustrated in Fig. 1(a), which encompasses acoustic monopole sources. The proposed enclosure (left panel) is a circular annulus with the outer and inner diameters *D* and *D*_*i*_ = 0.1*D*. It is made of a hard material (gray part), e.g. brass, for stark impedance mismatching to the filled air in the channels (white part). Therefore, the solid parts of the structure do not contribute in sound resonances and wave coupling, and in simulations the solid parts are legitimately treated as rigid boundaries^20,22^. Each coiled channel has width 0.03*D* and length 1.69*D* (red curve). Metamaterials with coiled channels were reported to have extreme properties^23^. Similar structures with coiled channels have been applied in achieving other novel phenomena in acoustics, such as double negativity, phase tunability, total reflection, and sensing^20,23–27^.

Here, the enclosure is adapted for generating resonances that are responsible for directivity enhancement. Note that the previous effective medium model simplifies a similar space-coiling structure as a homogeneous and isotropic medium^20^, which is different from our proposed effective medium model that accurately characterizes degenerate Mie resonances and the feature of inhomogeneity and anisotropy, as will be shown later. Additionally, a circular structure with coiled channels should have the radially dependent density as later evidenced by our derivation, which was missed in prior studies.

In the interior of the enclosure, point sources can be deployed at four locations denoted by the blue dots (P1, P2, P3, and P4) as shown in the right panel of Fig. 1(a). The sources are simulated with a constant source strength, i.e. a constant volume flow rate from sources, as a universal model^28,29^. In practice, an enclosure with *D* = 30 cm can accommodate cell phone transducers (∼3 mm) well inside its interior.

To demonstrate enhanced directivity by the enclosure, we choose P1 and P2 as two in-phase monopole sources, which are selected for optimizing constructive interference outside the enclosure. The simulation is done using the acoustic module preset in COMSOL^30^, without considering the dissipation effect (To reveal the essential physics, the simulation excludes dissipation. The dissipation effect will be considered in the latter case). When sources at P1 and P2 inside the enclosure radiate airborne sounds of wavelength *λ* = 1.14*D*, the acoustic pressure field (Fig. 1(b)) exhibits a unidirectionally collimated acoustic beam and its radiation pattern calculated in the far field (Fig. 1(c)) shows high directivity with minute sidelobes.

Contrarily, when the enclosure is removed, the far-field radiation pattern becomes omnidirectional (blue curve in Fig. 1(c)), indicating that the radiation is non-directive and that the sources radiate indistinguishably in the far field in free space. Note that our sound system that includes both the sources and the enclosure has an overall subwavelength scale, which is distinctive from other realizations that are composed of subwavelength elements but have the overall scale much larger than the working wavelength^7,18^.

Technically, to characterize radiation directivity, the polar plot for far-field directivity (Fig. 1(c)) is calculated by

1 $$F(\theta )={|{p}_{f}(\theta )|}^{2}/{\rm{\max }}\{{|{p}_{f}(\theta )|}^{2}\},$$

where ${p}_{f}(\theta )$ is the simulated far-field pressure. In Eq. (1) the square of ${p}_{f}(\theta )$ is proportional to radiated sound power, and *F* is a normalized function with respect to the azimuthal angle *θ*. To further describe the quality of the directivity, we define a quantitative indicator Δ:

2 $${\rm{\Delta }}=\,{\rm{\max }}\{{|{p}_{f}(\theta )|}^{2}\}/{\rm{mean}}\{{|{p}_{f}(\theta )|}^{2}\}\mathrm{.}$$

Equation (2) implies Δ = 1 for omnidirectional radiation and Δ > 1 for directive radiation. In the case where there is only one main lobe (a main lobe is the directivity lobe that connects $F(\theta )=1$ in a directivity polar plot), such as in Fig. 1(c), the narrower main lobe and the smaller side lobes make Δ larger.

**Figure 2:**
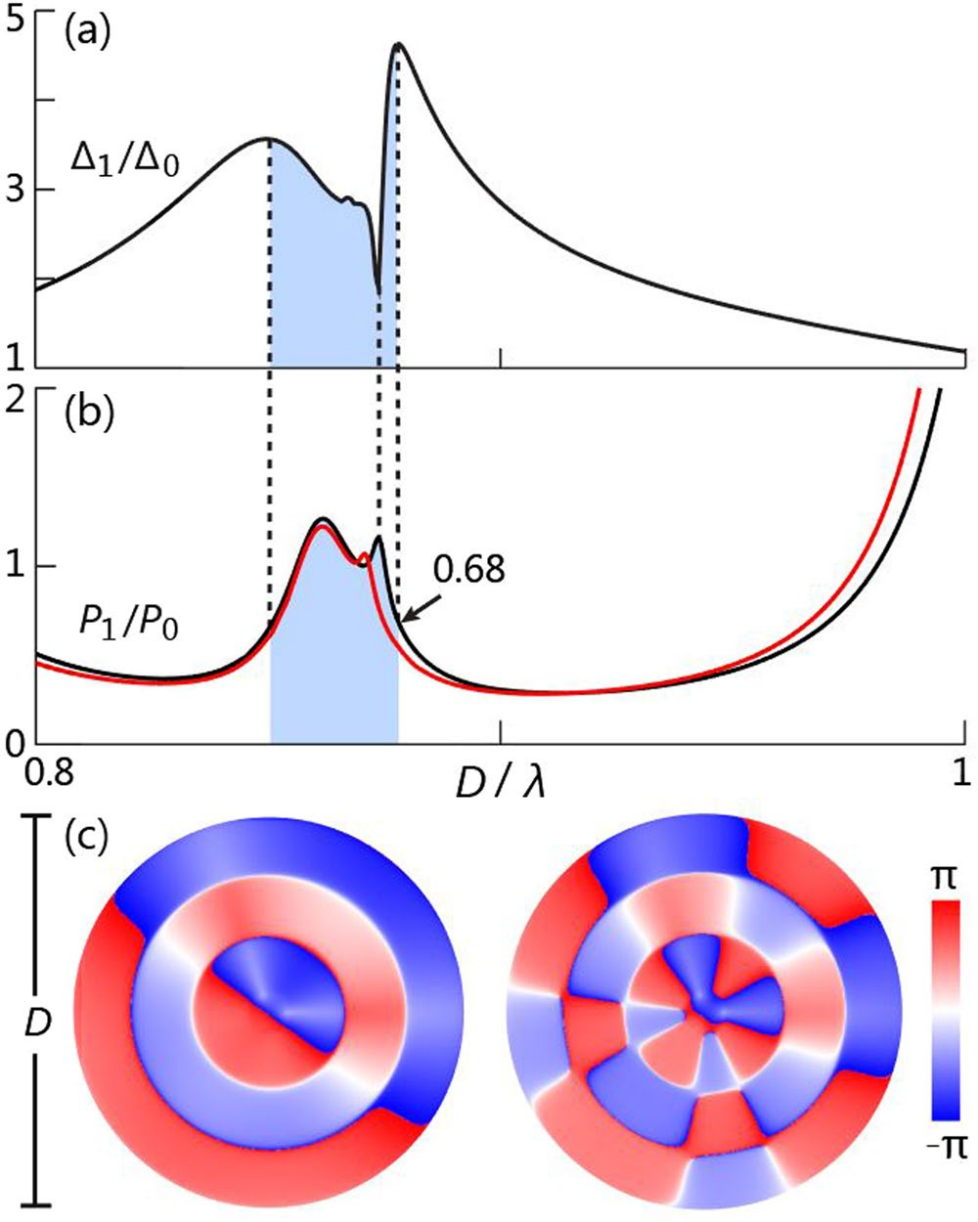
(**a**) Directivity enhancement Δ_1_/Δ_0_ for the in-phase sources at P1 and P2, where Δ_1_ (Δ_0_) is the directivity indicator with (without) the enclosure (See Eq. (2)). The local maxima and minima of Δ_1_/Δ_0_ are indicated by the vertical dashed lines. (**b**) *P*_1_/*P*_0_ is the ratio of radiated power, where *P*_1_ (*P*_0_) is the power with (without) the enclosure. In (**b**), the black curve is the simulation result in the case with the enclosure. *P*_1_/*P*_0_ is 0.68 when Δ_1_/Δ_0_ reaches maximum. The red curve is the simulation result with the enclosure’s effective medium. The two local peaks of *P*_1_/*P*_0_ happen at resonances, whose phase distribution is shown in (**c**).

Figure 2(a) shows the simulated directivity enhancement Δ_1_/Δ_0_ of the acoustic source layout (P1, P2) as a function of frequency, where Δ_0_ is the original radiation directivity in free space and Δ_1_ is the one with the presence of the enclosure. The maximum Δ_1_/Δ_0_ is found at *D*/*λ* = 0.88, and the corresponding acoustic pressure field and directivity polar plot have been presented in Fig. 1(b) and (c), respectively.

### Mechanism

The mechanism of directivity enhancement can be explained by investigating the ratio of the radiated sound power with and without the enclosure *P*_1_/*P*_0_ as frequency changes, which is indicated by the black curve in Fig. 2(b). The power is calculated through the integral over a closed surface in far fields. The results of the same quantity but with the enclosure replaced by its effective medium that has the parameters (The detailed derivation is discussed in the Methods Section).

3 $${c}_{r}=0.266{c}_{{\rm{air}}},\,{\rho }_{r}=25\pi {\rho }_{{\rm{air}}}r/D,\,{\rho }_{\theta }\to \infty $$

are plotted as the red curve in Fig. 2(b), which almost overlaps with the black curve. The effective *c*_*r*_ is derived from the conservation of the sound traveling time along the radial direction, and the effective *ρ*_*r*_ is derived from the impedance matching condition (See Section 4). The effective medium has the same size as that of the enclosure (${D}_{i}/2\le r\le D/2$). The effective parameters given in Eq. (3) characterize the anisotropic nature of the enclosure and are able to accurately predict the high-order resonances. Note that the previously derived effective medium model for a similar structure is isotropic and homogeneous, and its size is different from that of the original structure^18,20^. The effective medium theory for cylindrical coiled structures proposed here was not explored in other literature. Because of the popularity of coiled components in many acoustical applications, the accurate effective medium theory we propose could have a significant impact in guiding the future modeling of this type of the popular space-coiling structure.

To explore the physics clearly, the following analysis is based on simulations with the effective medium model replacing the real enclosure. At the first *P*_1_/*P*_0_ peak, the phase distribution in the region of the effective medium exhibits a dipole-dominant resonance profile (left panel of Fig. 2(c)), while at the second *P*_1_/*P*_0_ peak the phase distribution implies that the resonance is dominated by the octupole resonance (right panel of Fig. 2(c)). The simulated frequencies of these two dominant resonances at *D*/*λ* = 0.86 and 0.87 can also be theoretically predicted (0.86 and 0.87) based on the effective parameters given in Eq. (3), and therefore we are able to theoretically verify that the two dominant resonances indeed correspond to the Mie resonances of order 1 (dipole) and 4 (octupole), respectively. Additionally, in the region of the *P*_1_/*P*_0_ hump in Fig. 2(b), there could exist resonances of other orders with their magnitude being too weak to lead to local maxima of *P*_1_/*P*_0_. We attribute the small phase disturbance in Fig. 2(c) to the wave interference generated by the non-axisymmetric source layout.

Because the resonances of orders 1 and 4 (together with possible resonances of other orders) have the close resonant frequency, they cluster together to form a region of degenerate Mie resonances due to the enclosure’s anisotropy (the *P*_1_/*P*_0_ hump), where the superposition of multiple resonances occurs. The main consequence of degenerate Mie resonances is to enable simultaneous radiation enhancement of multipole modes of a monochromatic source in the subwavelength domain. Degenerate resonances could give a higher directivity because of interference between different orders of resonances.

Comparing Fig. 2(a) and (b), we find the maxima of the directivity indicator (Δ_1_/Δ_0_) are at the steep roll-off of the resonance region (left and right vertical dashed lines), and one Δ_1_/Δ_0_ dip occurs exactly at a *P*_1_/*P*_0_ peak (middle dashed line). At *P*_1_/*P*_0_ maxima, the entire enclosure works at strongest resonances due to the peak value of the radiated sound power. The strong sound field of *axisymmetric* amplitude induced by resonances at *P*_1_/*P*_0_ maxima “overwhelms” the interference field pattern caused by the *non-axisymmetric* source layout. Therefore, as the consequence of interferences being overwhelmed, Δ_1_/Δ_0_ does not reach maxima at the frequencies where *P*_1_/*P*_0_ maxima occur. Contrarily, when frequency moves close to roll-offs of the resonance region, the interference that accounts for non-axisymmetric radiation starts overpowering the resonance-induced pressure field of axisymmetric amplitude. Thus, the locations of the directivity peaks in Fig. 2(a) and those of the *P*_1_/*P*_0_ peaks in Fig. 2(b) are impossible to be aligned together, but could be close to each other.

The directivity enhancement for a unidirectional beam collimation has been demonstrated using an enclosure that encompasses in-phase sources at P1 and P2. Δ_1_/Δ_0_ reaches maximum 4.63 at *D*/*λ* = 0.86, with *P*_1_/*P*_0_ being 0.68. The value of *P*_1_/*P*_0_ at *D*/*λ* = 0.86 is low because the two sources are in phase and they can only significantly excite the resonance of the zeroth order at *D*/*λ* = 0.14, 0.43, 0.73, and 1.02, but not around 0.88 (The resonant frequencies for the proposed structure of an inhomogeneous density are determined by discretizing the enclosure into *N* homogeneous rings of different radial density and applying the general wave solution to each layer. There are in total 2(*N* + 1) unknown coefficients and unknown resonant frequencies forming an eigenvalue problem. It can be solved by scattering matrices formed from matching acoustic pressure and velocity at interfaces.). Multipole resonances that account for the *P*_1_/*P*_0_ hump around *D*/*λ* = 0.86 cannot be strongly excited by in-phase sources, and require non-uniform phase distributions along the azimuth.

### Bidirectional Beam Forming

**Figure 3:**
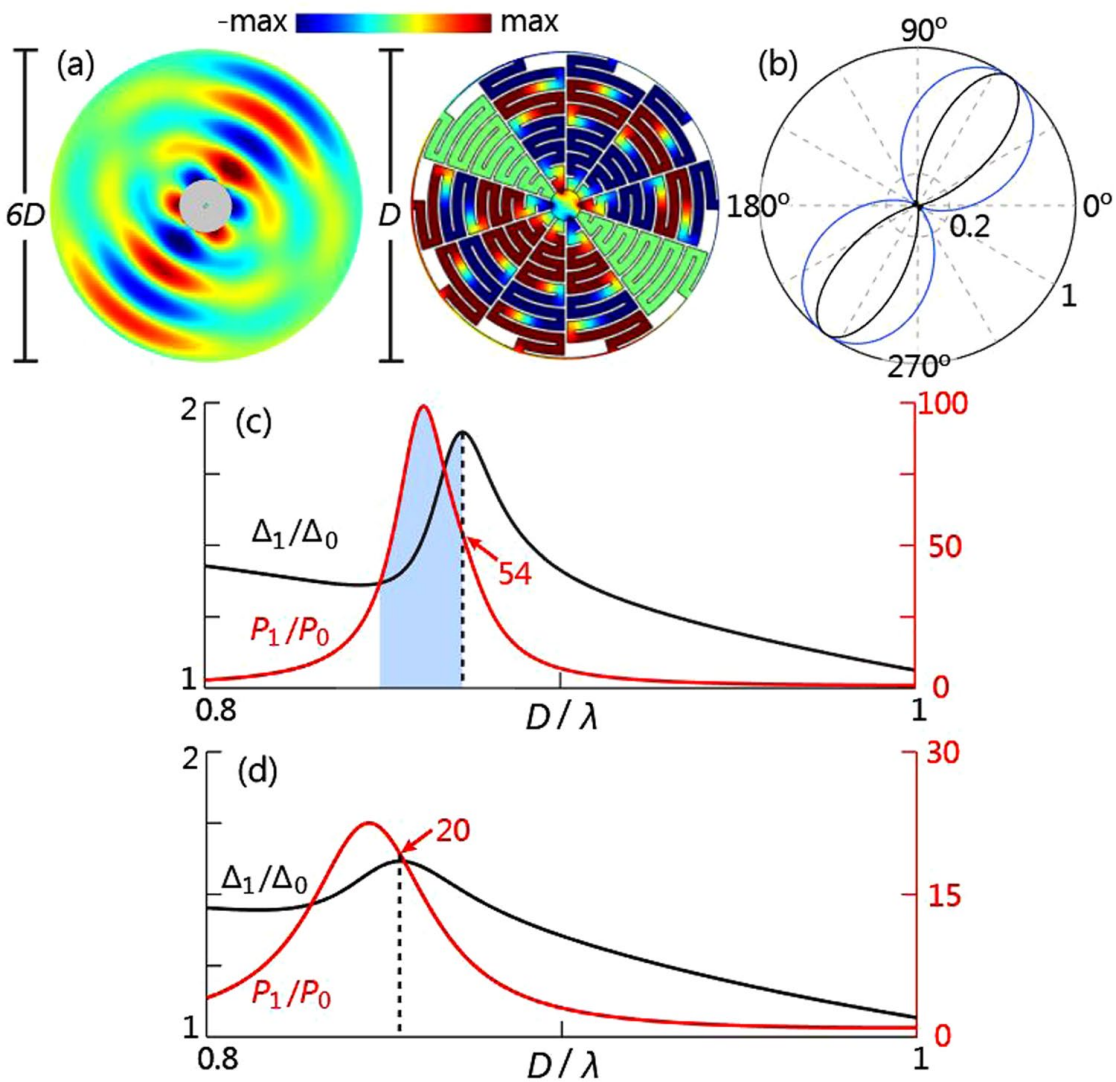
(**a**) With four sources encompassed by the enclosure, sound propagates bidirectionally with high directivity at wavelength 1.15*D*, as shown by the simulated acoustic pressure field. (**b**) The normalized far-field directivity with the enclosure (black curve) and without it (blue curve). The directivity maximum is enhanced 18.4 dB by the presence of the enclosure. (**c**) The directivity enhancement (Δ_1_/Δ_0_, black curve) and the enhanced source’s radiation rate (*P*_1_/*P*_0_, red curve). The maximum Δ_1_/Δ_0_ is indicated by the vertical dashed line, while its corresponding *P*_1_/*P*_0_ is 54. (**d**) Same as (**c**) but with viscous-thermal losses included.

To simultaneously enhance radiation directivity and source’s radiation rate, in addition to the in-phase sources deployed at P1 and P2 (same as in Figs 1 and 2), two sources, whose phases are opposite to those at P1 and P2, are deployed at locations P3 and P4. Such a four-sources profile gives rise to a bidirectional beam at *D*/*λ* = 0.87 (Fig. 3(a)). In free space, the four sources radiate indistinguishably and the directivity shows a standard dipole pattern (blue curve) in Fig. 3(b); however, with the enclosure employed, the two main lobes are narrowed without sidelobes, indicating strongly enhanced directivity (black curve).

In the subwavelength region *D*/*λ* ∈ [0.8, 1], the maximum directivity enhancement (black curve) is 1.90 at *D*/*λ* = 0.87, as shown in Fig. 3(c). Because the source layout in Fig. 3 is the extension of the layout in Fig. 2 by adding the specular out-of-phase sources, the frequency where Δ_1_/Δ_0_ reaches maximum remains the same, compared with the previous case. In Fig. 3(c), the *P*_1_/*P*_0_ hump (red curve) also results from the superposition of degenerate multipole resonances of different orders. The *P*_1_/*P*_0_ maximum is contributed dominantly from dipole resonance, because of the mirror layout of the out-of-phase sources. Here, the enhancement of sources’ radiation rate *P*_1_/*P*_0_ is remarkable, because the source layout that includes out-of-phase sources significantly excites degenerate multipole resonances around *D*/*λ* = 0.87.

In Fig. 3(c), still at the steep roll-off of the resonance region (*D*/*λ* = 0.87; vertical dashed line) Δ_1_/Δ_0_ reaches maximum, and simultaneously *P*_1_/*P*_0_ hits 54. To further explore the influence of thermo-viscous dissipation, we use COMSOL for simulations, whose robustness has been numerically and experimentally verified previously^22^. Specifically, we used the interface coupling between the embedded pressure acoustic module and the embedded thermo-acoustic module for simulations with losses included. Fine mesh was applied to the proximity of the air-structure boundaries to resolve the thin thermo-viscous layers. The diameter of the enclosure is chosen *D* = 10 cm, the solid body is treated as acoustically rigid in simulations, and the simulated results are shown in Fig. 3(d). We still notice the strong enhancement of both radiation directivity and radiation rate, and the proposed enclosure remains its subwavelength scale.

## Discussion

In conclusion, we present to use a space-coiling enclosure and its accurate effective medium model to achieve beam collimation with high directivity, and the entire sound system remains subwavelength. Inside the enclosure, sources are configurable to achieve high directivity in the far field, though they are otherwise indistinguishable in free space. We demonstrate unidirectional and bidirectional beam collimation. The directivity enhancement occurs at the roll-offs of the region of degenerate Mie resonances. This study provides a solution to the challenging problem in acoustics: acoustic sources at low frequencies are the radiators with poor radiation directivity in free space. The endeavor of enhancing sources’ radiation directivity only by a subwavelength enclosure as a configurable subwavelength sound system could have an impact in acoustic device miniaturization. Additionally, for the circular space-coiling enclosure that has zigzag channels, we provide an effective medium model that has the size equal to the original enclosure and characterizes the enclosure’s inhomogeneity and anisotropy.

## Methods

### Effective medium theory for the space-coiling enclosure

**Figure 4:**
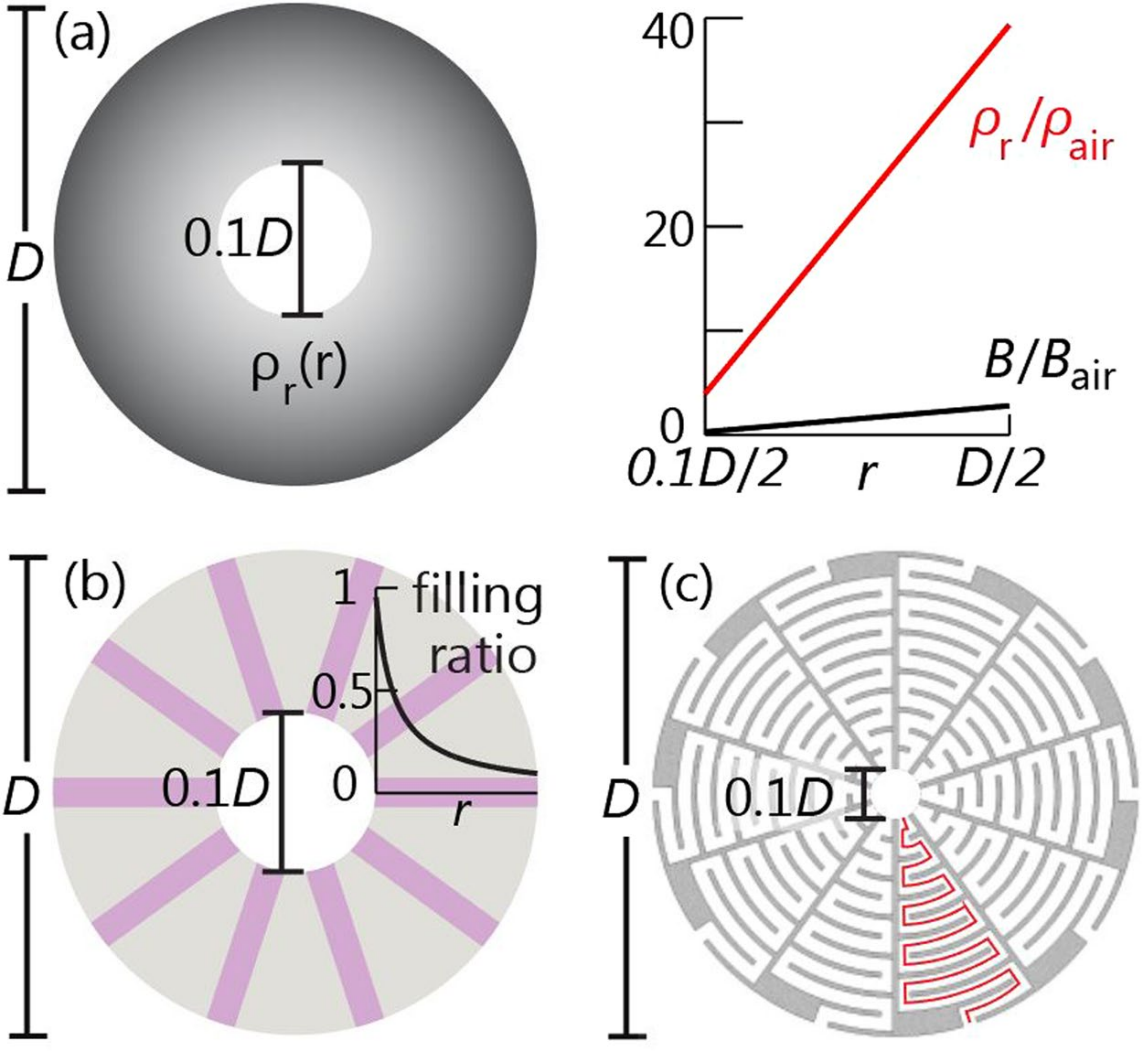
(**a**) An abstract medium (left panel) with inner and outer diameters 0.1*D* and *D* has an infinite azimuthal density *ρ*_*θ*_ and a constant radial sound speed lower than the sound speed of air (*c*_*r*_ << *c*_air_). It has radially dependent radial density *ρ*_*r*_ and bulk modulus *B*, as shown in the right panel. (**b**) The preliminary equivalent model made of rigid materials (gray) with straight channels (purple) has the radially varying filling ratio. (**c**) The enclosure made of rigid materials (gray) has air-filled channels with coiled sound paths (red curve).

Here we verify that the abstract medium shown in Fig. 4(a), which has the inner diameter *D*_*i*_, the outer one *D*, and the acoustic parameters

4 $${\rho }_{\theta }\to \infty ;{\rho }_{r}=25\pi {\rho }_{{\rm{air}}}r/D;{c}_{r}={c}_{{\rm{air}}}/n;n=\mathrm{1/0.266,}$$

is an accurate effective model for the enclosure shown in Figs 1a, 4c. The equations was given briefly^19^ and here we provide a detailed derivation.

As the first step, we equate the abstract medium in Fig. 4(a) with the preliminary equivalent model shown in Fig. 4(b). The preliminary model is made of brass (gray part, density *ρ*_brass_ = 8500 kg/m^3^; bulk modulus *K*_brass_ = 1.88 × 10^11^ Pa), and has the inner and outer diameters (*D*_*i*_ and *D*) same as those of the abstract medium. It contains *M* straight channels (purple) of width *w*. We assume all channels are filled with a certain fluid having constant density *ρ*_m_ and constant sound speed *c*_m_ to be determined. The radial filling ratio of the straight channels varies along *r* according to

5 $$q(r)=Mw/\mathrm{(2}\pi r),$$

illustrated in the inset of Fig. 4(b).

To make the preliminary model in Fig. 4(b) have the material properties same as those of the abstract medium in Fig. 4(a), we assume the specific acoustic impedance in the purple straight channels much smaller than that of brass. Because the brass parts can be treated as rigid bodies in this case, the preliminary model has *ρ*_*θ*_ → ∞. Additionally, based on the effective medium theory for acoustic gratings^31–33^, we reveal the condition that makes the preliminary model have the material properties same as those of the abstract medium:

6 $${c}_{{\rm{m}}}={c}_{r};\,{\rho }_{{\rm{m}}}=q(r){\rho }_{r}\mathrm{.}$$

Because *ρ*_*r*_ in Eq. (4) is proportional to *r* while *q* in Eq. (5) is inversely proportional to *r*, both *ρ*_m_ and *c*_m_ are constant, coinciding with our earlier assumptions.

Next, we equate the preliminary model in Fig. 4(b) with the enclosure having air-filled channels in Fig. 4(c). The enclosure has the size *D*_*i*_ and *D* same as those of the abstract medium. It is made of brass (gray parts) that separates *M* coiled channels. Each coiled channel of width *w* (same as that of the preliminary model) has a sound path *L* to be determined (red curve).

We find the conditions for equating the preliminary model with the enclosure are:

7 $$\begin{array}{c}{c}_{{\rm{m}}}{\rho }_{{\rm{m}}}={c}_{{\rm{air}}}{\rho }_{{\rm{air}}}\\ (D-{D}_{i}\mathrm{)/(2}{c}_{{\rm{m}}})=L/{c}_{{\rm{air}}}\end{array}\mathrm{.}$$

The first condition dictates that the acoustic impedance in the coiled channels in Fig. 4(c) should be the same as that in the straight channels in Fig. 4(b). The second dictates that the sound-traveling time from the inner rim to the outer rim should remain unchanged. The first condition also implies the acoustic impedance of the straight channels in Fig. 4(b) is 9.6 × 10^4^ times smaller than that of the brass walls, fulfilling the earlier assumption for *ρ*_*θ*_ → ∞.

Plugging Eqs (5) and (6) in Eq. (7), we derive the parametric dependence of *ρ*_*θ*_, *ρ*_*r*_, and *c*_*r*_ on *M*, *w*, and *L* of the enclosure:

8 $${\rho }_{\theta }\to \infty ;\,{\rho }_{r}=\frac{4\pi L{\rho }_{{\rm{air}}}r}{Mw(D-{D}_{i})};\,{c}_{r}=\frac{(D-{D}_{i}){c}_{{\rm{air}}}}{2L}\mathrm{.}$$

For a given set of *ρ*_*r*_ and *c*_*r*_ such as in Eq. (3), we can determine *M*, *w*, and *L* according to Eq. (8) so that the abstract medium in Fig. 4(a) and the enclosure in Fig. 4(c) have the same material properties. The enclosure shows exactly how it looks like for a specific set: *M* = 10, *w* = 0.03*D*, and *L* = 1.69*D*.
