# Author Correction: Directional sound beam emission from a configurable compact multi-source system

**DOI:** 10.1038/s41598-018-35523-z

**Published:** 2018-11-21

**Authors:** Jiajun Zhao, Rasha Al Jahdali, Likun Zhang, Ying Wu

**Affiliations:** 1 King Abdullah University of Science and Technology (KAUST), Division of Computer, Electrical and Mathematical Sciences and Engineering, Thuwal, 23955-6900 Saudi Arabia; 2 GOWell International LLC, Houston, Texas 77041 USA; 3 National Center for Physical Acoustics and Department of Physics and Astronomy, University of Mississippi, University, Mississippi, 38677 USA

Correction to: *Scientific Reports* 10.1038/s41598-017-16792-6, published online 18 January 2018

The original version of this Article contained a typographical error in the spelling of the author Rasha Al Jahdali, which was incorrectly given as Rasha Al Jadhali. This has now been corrected in the PDF and HTML versions of the Article.

